# Dental Antiseptics and Disinfectants

**Published:** 1901-10-15

**Authors:** Byron L. Kesler


					﻿THE
DENTAL REGISTER.
Vol. EV.	October 15. 1901.	Xo. 10.
DENTAL ANTISEPTICS AND DISINFECTANTS.
BY BYRON E. KESEER, D.D.S.
Abstract of Thesis presented by Dr. Kesler for the degree of Doctor of Dental
Science at the University of Michigan.
Before considering the subject in detail, also to aid
in a more clear understanding of the topics treated in
the present paper a definition of the terms involved mav
be in order.
An antiseptic is an agent which inhibits the growth
or development of micro-organisms.
A germicide kills the organisms. If sufficiently
diluted, it may then act as an antiseptic.
Sterilization means the complete destruction of all
micro-organisms and spores present.
Disinfection means the partial sterilization, this
being the usual significance of the term. Disinfection,
usually, means the destruction of the bacterium (and
its product), which is producing the disease, and mav
or may not destroy other organisms which may be
present.
Antiseptics act in two ways. First upon the
tissues or substances to be sterilized. . Forming a union
with the tissues of such a nature, that the agent is re-
tained, for a period of time, more or less definite,
depending upon the character of the agent used.
Secondly, the antiseptic must be in solution when used,
or else soluble in the tissue fluids. Bacteria are
unicellular-protoplasmic organisms, enclosed in a mem-
brane, composed of the same material, in condensed
form. Sterilizing agents must be in solution, to enable
them to pass into the cell by diffusion through the
enveloping membrane.
To be efficient, an antiseptic must be of such a
character, that it will react upon the organisms. But
of such character as not to be too easily dissipated by
body fluids. This principle is very well illustrated in
the action of Iodoform. When in the dry form, or
powder, bacteria may be found growing and developing
in the iodoform bottle, but when the iodoform is in
solution, no organisms develop.
The principle is again well illustrated in the action
of gases, especially the fames of sulphur. When the
sulphur dioxide (SO2) gas is used in a dry state it is
not an effective germicide, though if the materials or
walls of the room to be disinfected, be sprayed with
water before the gas is applied, then a complete steri-
lization may be affected.
Antiseptics need not necessarily be germicides in
order to be efficient terapeutic agents.
DISINFECTANTS.
These may be either general or specific in their
action. A general disinfectant is one which may de-
stroy all the organisms, pathogenic and non-pathogenic,
while a specific disinfectant destroys the organism (and
its product), which is producing the disease, and may
or may not destroy others which may be present.
This principle being well illustrated in the use of
Antitoxin, and the antitoxic and antiinfectious serums.
Bacteria are specific in their power of resist-
ance, being more susceptible to some disinfectants than
to others. Again, one species, being more hardy
and resistant than another, they are also differently
resistant to physical agents, heat, cold, electricity, etc.
As to heat, the character of which will make a marked
difference, whether it be moist or dry, this feature is
determined by proper methods in the laboratory. The
‘•Thermal Death Point,” being accurately ascertained,
not only as to the degree of temperature, and the time of
exposure required, but the different species of bacteria
may also be tested. The relative specific resistance,
being determined, we are now ready to demonstrate the·
action of disinfectants and antiseptics.
These agents may be tested in two different ways,
or two methods are employed.
First of all a Bacterial “ Suspension ” is prepared,
as follows : A good active culture, of the organism to
be tested, is grown upon inclined Agar. Sterilized
water (by autoclaving), is then introduced into the
Agar tube, by means of a sterile pipette, and well mixed
with the growth (upon the surface of the Agar). This
aqueous solution is then transferred with sterile, bulb
pipette, to a specially prepared, cotton and glass-wool
filter (also sterilized), and received into a sterile glass
dish. The filtering segregates the bacteria, giving the
disinfectant a better opportunity to reach or act upon
all of them.
METHOD A.
“ Specimens ” a,re prepared, small squares (i inch)
of muslin, short silk threads (A inch), and small glass
squares (4 inch). These are sterilized by dry heat.
When cool, the “specimens” are soaked in the bac-
terial “ suspension,” dried and then immersed in the
disinfectant solution. At stated intervals, with sterile
forceps, one each of the specimens is transferred to a
tube of sterile boullion, properly labelled and placed in
the incubator to develop.
EXPERIMENTS.
Experiments were made with both moist and dry
heat to show the relative disinfectant properties. Those
of the most resistant micro-organisms were used in the
experiments with the square of muslin, thread and
glass as vehicles.
With the moist heat at a temperature of 58° C
the bacilli of Cholera, Green Pus and Sanarelli were
exposed for various times from one minute to sixty and
examined after three days and all found sterile. After
four days the one minute cholera showed growth and
the five, ten, and fifteen minute sanarelli showed growth,
the forty-five minute showed no growth after four days.
At 70° the results were the same for three days, but
the one, three and five minute cholera showed growth
after four days. At too0 all were sterile and remained so.
With the dry heat at 70° using sanarelli only,
after two days there was a growth after exposures for all
times including sixty minutes. At ioo° there was
sterility after forty-five and sixty minute exposures.
At 1200 there was sterility after thirty minute exposures.
At 150° there was sterility at and after five minute
exposures.
METHOD B.
The disinfectant solution and bacterial “suspension"
are mixed, in equal volumes of each. The time is
taken, and at stated intervals, one drop is transferred
to a tube of sterile boullion. The disinfectant is thus
diluted one half and the calculations must be made
accordingly.
When a drop of the mixture is transferred to the
boullion, enough of the drug may be “ carried over,”
to act as an antiseptic in inhibiting the development oi
the organism, already attenuated by the exposure to the
medicine. To overcome this objection the drop of mix-
ture is transferred to a generous amount of boullion,
say ioc. and thoroughly mixed.
In all of these tests “ control ” tubes are placed in
the incubator. One tube is innoculated with the bac-
terial suspension, before it is mixed with the disinfectant.
Other tubes of plain boullion are used. These “control”
tubes test the vitality of the organism in the suspension,
after filtration, also the effect of incubator temperature
upon the boullion, as to sterility, also physical change.
Thus fair and accurate determinations can be made.
In these tests we ascertain the resistant power oi
bacteria to disinfectants in general, also their specific
resisting power to certain drugs. The following tables
taken from Prof. Novy’s demonstrations, will explain
the principles involved, and show the relative power
of hydrochloric and sulphuric acids on different germs.
II. Cl. Mineral Acids. II2 SO4
.099	Anthrax	0.07
.014	Diphtheria	0.20
.050	Glanders	0.50
.330	Typhoid	0.20
.070	Cholera	0.07
The principle of chemical solubility also plays
an important part. Bichloride of Mercury, when
brought into contact with albumin will precipitate as
the albuminate of mercury. And in this condition is
inert as a germicide. The precipitate, however is
soluble in an excess of albumin.
Again, mercury is precipitated from its salts by
hydrogen sulphide.
Laboratory experiments—I. Add Mercuric Chlor-
ide to Anthrax Culture and we get no growth. 2. At
certain periods, pass a stream of hydrogen sulphide
through the boullion or culture, mercury is precipitated
as sulphide. Now transfer a few drops of the culture
to a fresh tube of boullion and development will occur
(up to a certain time). The same results are demon-
strated when the Anthrax culture, after treatment, be
injected into a guinea pig.
These experiments demonstrate that bichloride is
not a good disinfectant to use in supprative conditions.
Where tissue decomposition is actively progressing and
hydrogen sulphide is being liberated, Phenol, however,
is more effective in these cases.
By experimentation, with boullion, oil and aqueous
“ suspensions,” we learn that the character of the
culture media influences the effect or penetrating power
of the disinfectant. Boullion and oils seem to protect
the bacteria from the action of the drug used.
For this reason, body fluids may exert an entirely
different influence upon drugs, than is manifested in
laboratory experiments when conducted in aqueous
solutions.
♦
In making the following tests with the disin-
fectants, upon bacteria, I used aqueous suspen-
sions in each case, then made the transfers to boullion.
Conditions were made as uniform as possible, from the
results obtained, we observe, that with Pyocyaneus,
the bacillus of green pus, Phenol five percent germi-
cidal at ten minutes ; bacillus of green pus, Creosote five
percent germicidal at one minute ; bacillus of green pus,
DISINFECTANTS.—METHOD B.
Aqueous filtered, Bacterial “ suspension ”disinfectant equal vol-
umes of each at the end of stated periods one drop of the mixture
transferred to Boullion tubes then placed in incubator.
Exa- TIME OF EXPOSURE JO DISINFECTANT.
Percent.j	mined —j j g | 3 I 5 I 10 I 15 I 30
uays Min. Min. Min. Min. Min. Min. Min.
5	PhenolTB. G. Pus..	1J.	2p	Zp	Zp
1-1000 Hy. Clr.+Sanarelli. J
10	Lysol-|-B. G. Pus...	2	4-	4~	4~	4 1-—
5	Lysol-j-B. G. Pus...	2	—	’—	—
1	Creosote-1 B. G. Pm 3	4~	4-	+	4-	4
1	Creosote-j-Sanarelli 3	-;-	4~	+	4j	4~
5 Creosote4-B. G. Pus | 9	__ _________ _____ ____ ____ _____
¡13	_	_	_	_	_
5	Creosote4-Sanarelli	15	—	—	—	—	—
2	Creosote4-Saliva	I..	4	—	—	—	—	—	—
5	Lysol-j-Sanarelli....	6	—	—	—	—	—	—-
2	Lysol4 Sanarelli	...	5	—	—	—	—	—	—
2 Lysol-|-B. G. Pus...	~	__
2 Lysol-pB. L. Acid.. / g ______	__ ____ _____ ____ ____
9J Milk of Magnesia-]-
~'2 Py. Aureus........	5	4“	4-	4~	4-	4-	+
2 Lysol-f-Saliva 1.... /	__ j	_	__ _____ ____ ____
2 Lysol-f-Py. Aureus. {-	__ __________ ~	__ ____
2} Lysol4~Py· Aureus. 7-4	4----—	—	—
91 Borolyptol-|-Py.
Aureus.......... 5	-f-
Borolyptol4-Py.	12	4-
Aureus......... f	4	-j- ,
100	Borolyptol-·-	J	1
Py. Aureus.... (3
,nn	Eucalyptol4-	J 3	_p	__	_	_
Py. Aureus.... I 6	-j-	—	—	—■
20 Milk of Magnesia-p
Py. Aureus....
Creosote one percent not germicidal at all. Bacillus
from Saliva, Creosote two percent (saliva), germicidal
at one minute ; bacillus from Saliva, Lysol two percent
is a germicidal at one minute.
For some of these tests I isolated micro-organisms
from the saliva, bacillus of green pus, Lysol two per-
cent is germicidal at once, one minute ; bacillus ot
Saliva, Lysol two percent is germicidal at once, one
minute ; pyogenes Aureus (pus coccus),Lysol five-tenths
percent is germicidal at once, one minute ; bacillus of
Lactic acid, Lysol two percent is germicidal at once,
one minute ; Pyogenes Aureus, Eucalyptol fifty percent
is germicidal at once, one minute ; Pyogenes Aureus,
Borolyptol fifty percent not germicidal at all, several
experiments.
In each case of the above table it should be noted
that the solution indicated of the disinfectant in the first
column was twice as dilute after it had been added to
the suspension liquid.
We observe that the homologues of Phenol (Cresol,
etc.), also the saponified form, or emulsion (Lysol).
are effective at once, while Phenol, purified, requires
time. We may also note with profit that Lysol two
percent is germicidal at once, to the pus organisms, to
the bacillus of Lactic Acid ( which produces caries ),
and to the organisms isolated from the saliva.
ANTISEPTICS.
With these agents we test their power to inhibit and
prevent growth of micro-organisms. The method of
operating is as follows : A one percent solution of the
reagent is made, and steamed a few minutes. Sterile
boullion in bulk, sufficient for the experiment, is in-
noculated from a boullion culture and well mixed.
Then the required amount is transferred by means of a
sterile, graduated pipette, to a sterile empty tube. The
calculated amount of the reagent is then added by
means of another sterile, graduated pipette.
Phenol i % solution—2cc See suspension¡500
1	“	9 “	“	= 1	:1000
0.5	“	4-	9.5 “	“	=1	:2ooo
0.25“ +9-75“	“	=1:4000
0.1“	4-	9.9 “	“	-i	: 10000
Bichloride 1 1500—.1	“	4-	9.9“	“	I	:5OOO°
The tubes are then properly labelled and placed in
the incubator, and with them, the “control” tubes
also. (In some instances, as in testing oils, the oil is
placed in the bottom of the tube first, and the innocu-
lated boullion is added subsequently. The oil then
diffuses upwards through the boullion) .
In twenty-four hours the tubes are examined. From
those in which no growth is discernible, a few drops
are “ transferred” to a fresh tube of boullion, labelled
and placed in the incubator to develop. Several days,
or a week is allotted, to insure accuracy and to eliminate
possible error, from too hasty an examination, also to
allow the germs time to develop. From experiments
made in this way we derived the following results :
. Bichloride is antiseptic at 1 to 50,000
Formalin is antiseptic at 4 to 10,000
As to Pus organisms
Phenol stands antiseptic at 1 to 10,000
Lysol stands very near 1 to 10,000
Creosote stands at	1 to 2,000
Thymol stands at about 1 to 3,000
With Strepto-Coccus, Thymol, between 1 to 2000 and
1 to 1500; with Strepto-Coccus, Boro Lvptol, between
1 to 2000 and 1 to 1500 ; with Pyogenes Aureus, Oil
Cinnamon, between 1 to 2000 and 1 to 1500; with Py-
ogenes Aureus, Boro Lyptol, between 500 to 2000
attenuated, not killed ; with Pyogenes Aureus, Eucal-
yptol, did not inhibit at I .-500 ; with Pyogenes Aureus,
Milk of Magnesia, antiseptic at 2 to 4000 ; with Strepto
Coccus, Milk of Magnesia, antiseptic at 2 to 4,000 or
i to 3,000.
Comparing Phenol with Creosote, we find that as
an antiseptic Phenol is superior, but as a disinfectant or
germicide that Creosote is more effective, two percent
Creosote being stronger than five percent Phenol ; and
as a germicide, Lysol is superior to either of them,
half of one percent Lysol being stronger than five per-
cent Phenol, or one percent Creosote.
ANTISEPTICS.	TRANSFERS.
•O	13
(D	o
.£	.£
S 2	O	1 «5	o
x3	O	O	O	O	x	0000
HJ® O O	O	O	O	LUO ° OOOO
00000	“ o OOOO
Ï in w- c\j	®	m	CM	_
I	I	I	I	I E	I	'III
”	W-	_ I _ I _
(	24	—	_ ___ ___ __ I
FormalinySanarelli.. 1	_ ___ ________ _ _ 3	—	— — —	-]*
Phenol—Sanarelli { 36   i +
Hg. C12—Sanarelli.... {	^4	— ; —	—	—	—	3	1-5W00
PhenolyPy. Aureus. 24	—	—	—	—	—	4	1-500	—	—	—	—
Creosote—Py. Aureus...	24	—	—-	4-y	y	M	:	—	—	-j-	y	y
Lysol Py. Aureus.. 24	—	—	—	—	—	4	—	—	—
Lysol—Strepto Py.. 24	—	—	—	—	—	4
ThymoiyPy. Aureus....	72	—	—	—	y	y	3	y	y	y
Boro Lyptoiy Strepto Py	—	—	+	+	+	'	—	—	+
Milk of Magnesiay
Strepto Py.	48	—	—	—7	— 1	—	4	—	—	— y
Oil of Cinnamony	5	—	—	—
Py. Aureus.	—	—	y	y	y	4	—	—
Oil of Cinnamony f	96	—	—	—	—	—	y
Py. Aureus. 1	72 —	— ; —	— — I
Boro Lyptoly
Py. Aureup. ¡4----i y-— -■	' ■	I y
Milk of Magnesiay
Py. Aureus.	48 —	— , —	y y	i	y 4- y y i y
Eucalyptold-Py. Aureus y	4- y	y -	3
I	i i I	1
Creosote, a homologue of Phenol, is more of an
alcohol, it contains a methyl (CH3) group in addition
to the hydroxide (OH) or Phenol group. “The hy-
drogen in the (OH) group prevents the methyl group
from being oxidized.”, (Dr. Prescot).
Comparing Lysol with Phenol and Cresol, we ob-
serve that Lysol is much stronger than either of them.
With the one exception in favor of Phenol, as to its
specific action upon Pus Cocci.
As our knowledge of bacteriological science devel-
ops, we observe this important fact, that each species
of bacteria is more susceptible, or less resistant to some
drugs than to others. In relation to Phenol, this obser-
vation should be noted. That Phenol, while being a
most pronounced germicide upon some organisms, it is
itself, in some cases a bacterial product. Phenol is
being produced all the time in the intestines of animals,
including man, by putrefactive bacteria. The time is
approaching when we will be enabled to select medi-
cines for germicidal purposes with as much accuracy
and precision as is now done with reference to their
chemical and physiological action.
Inorganic substances when combined with an or-
ganic group, become very much more intense in
germicidal power. (Again I quote Prof. Novy.)
crude, Phenol is much more powerful than the re-
fined Phenol, the enhanced power is derived from the
organic group and the Cresols. The Cresols are
less soluble then Phenol, but when brought into solution
by means of an organic acid or its salt, it then becomes
much more active than Phenol.
Saponified Phenol in an emulsion, as is repre-
sented by Lysol, is ten times stronger than Phenol.
Lysol is more general in its application, than is Carbolic,
and can be more freely used, as it is not so destructive
to animal tissues, at the same time being highly poison-
ous to bacteria.
Essential oils are not soluble in water. They are
therefore used in alcoholic solution, a portion of the
germicidal power is thus due to the alcohol. When
these oils are suspended over bacterial cultures, the
vapors will kill the organisms. This fact may be
applied to the treatment of root canals. V aporizing
the essential oil with hot air or a metallic root canal
dryer.
“ An aqueous solution of essential oils may be pre-
pared by saturating the cotton-wool in a filter, and then
filtering water through the oiled cotton.”
COAGULATION OF ALBUMIN.
The following experiments were made to determine
the property of medicines to coagulate albumin :
Fresh egg-albumin is cut up with scissors or knife.
Then diluted with sterile water. This diluting with
water makes the test more delicate, also prevents the
albumin from being coagulated or changed in any wav
bv the action of heat. The diluted albumin is then in-
noculated with a bacterial “suspension” and filtered.
In these experiments I used the strepto-coccus-pyo-
genes. The most resistant of the pus organisms. The
mixture was then drawn up into capillary pipettes, (of
uniform size), and the lower end of the tube sealed in
the flame.
The cotton plug at the upper end of the tube is then
removed and the medicine (in uniform amounts) intro-
duced. The tube re-pluged, labeled and placed in a
perpendicular position in a suitable rack. Control
tubes of the albumin before the medicine is added, are
TABULAR STATEMENT OF COAGULATION.
Egg Albumin 17 percent—Strepto-Coccus Pyogenes.
3	i 2	' rS
.2	75	&
•	2 y al	u	<v	¿5 ’S
C	-5	c	bo a 4? ö	= -H
?	·-	bn	«	<D	i	c	«	c3	*-<	Ä	r~*
Sh	^7	—	CO	C	i	CO	O	·	73	<D	M	-*-*	O
CD	■	o	·—'	«-H	1	·—<	r—i	·"*	r—i	«1-1	O
^5	o Q EH I o______________Q o________O____O______________E-<
80|SNiVtrrate + ’	8 in’ hrs. mi,lti 8	3 in' 11 in’
80 5°° -j + +	13“ at	white	13	13
Chlorid	once
1:1000	Chlorid	hrs.	lower	8	upPer 2in.
(Hg)	“	™	~r	10
SOiAlcohol	+	|	“	4— 2-3“	4-	13	Ki
951Ä	+	*“	“	+ sj	+-	n	15
back
5|Phenol P"™ 10	“ wall 10	10
'	white
95 Phenol 4- 4- 3|4-5’, “ white	middle	10
011-	(( gray	|	in the	9 .	., ;
100 Creosote ' ,	' 4,' P	white	1	middle in' -
4-	1 '»· y_gray
40 Lysol	“ capil- 5 1	attop 51	101
lary
j00 Black’s	« y^g> 5	9
white	all
40Form1a.-	+'+way	9
On	gJ
48
i nn Oil of	hrs. 4-----4	1	,	10
Cassia	72 y-gray 10	10
hrs.
100 °2’	48 y-gray 4-3	4-	6	9
j Sanitas	J J
J0O Enca-	_	« y. brown 4,	,	41	9
lyptol	necks
33 Gallic .	.	« y.brown 7	+	4	11
Acid	Hecks
33 Tannic	,	« white n	11
Acid	'
f 48
Control ------- q 7g none j
made. And also a few drops of the innoculated albumin
are transferred to boullion, to act as “control” for the
vitality of the organism in the albumin. These tubes,
rack and all, were then placed in the incubator.
The tubes were examined at frequent intervals, to
note coagulation, measurements were made and results
recorded. When satisfied as to coagulability, after sev-
eral days, transfers were made to boullion in the follow-
ing manner : A file mark was made on the tube where
we wished to make the separation. It was then im-
mersed in bichloride up to the mark and some distance
beyond. With sterile forceps the tube was then broken
off and the contents of the fragment transferred to boul-
lion. The tubes were properly labeled and placed in
the incubator.
From the results of these experiments several de-
ductions can be made and we are enabled to make the
following comparisons :
Zinc chlorid coagulates at once, and completely.
Bichloride an incomplete coagulation, two to six-
inches, the balance of the tube being changed in color
only—bluish white.
With phenol 95 percent, a curious phenomenon oc-
curred. There was a complete coagulum for a short
distance, then a clear space, succeeded by coagulation
again.
Phenol 5 percent. K partial coagulum formed
throughout the whole tube. A film of white coagulum
formed on the back wall of the tube.
Creosote acts very similar to dilute phenol.
Black's 1-2-3. Not a complete coagulum at any
point, but there was a change of color the whole length
of the tube, probably due to the oils.
Lysol, a yellowish-gray color for a short distance,
then a change of color similar in appearance to Black’s
i-2-3·
Formalin. No complete coagulum at any point,
though a change of color, bluish-white, similar to the
silver nitrate terminal.
Tannic acid coagulates· completely and extensively.
Gallic acid. A partial coagulation five to seven
inches, a change of color two to three inches, the ter-
minal remaining clear.
Essential oils. No coagulum, only a change of
color.
TRANSFERS TO BOULLION.
In this experiment several good opportunities pre-
sented for testing the germicidal property of drugs, be-
yond the point of coagulation, especially the alcohols
and gallic acid. In each instance there was a coagu-
lum in the upper portion of the tube, and the terminal
of the capillary remaining clear. A distance of five or
six inches intervening. Yet when these terminals were
transferred to Boullion, no growth developed in either
case.
The essential oils, silver nitrate, lysol, formalin and
bichlorid, were not coagulated in the terminals, though
a change of color was present, these when transferred
to boullion, gave no development.
Phenol 5%, is the onlv one which gave a growth.
This was decisive and no question about it (several
trials or tests being made.) Referring again to phenol
as a disinfectant, we find that with the bacillus of green
pus, which is a much weaker germ than strepto-c.occus,
(the latter being used in these coagulation experiments)
a growth was developed in the exposures of one, three
and five minutes. Again in the antiseptic tests, phenol
was not antiseptic to the bacillus sanarelli at any pro-
portion (i : 500 or greater). Sanarelli (the bacillus of
yellow fever) is very similar to the strepto-coccus pyo-
genes in resisting power.
These coagulation experiments determine two
things : the relative coagulability of the medicines used
in dentistry, also, that not only where actual coagulation
exists, but beyond that point the medicines are germici-
dal. Thus refuting some statements made to the con-
trary ,
A tooth, in situ, is connected to a circulating medi-
um, which is also germicidal. The phagocytes of the
blood also have the property of secreting a highly toxic
substance, neuclenic acid.
As demonstrated by laboratory experiments, the
vapors of medicines are germicidal. Then why not by
diffusion through the coagulum ? These experiments
seem to determine beyond question, that coagulants
have a germicidal value beyond the point of coagula-
tion.
				

